# Biology, Genetics, and Environment: Underlying Factors Influencing Alcohol Metabolism

**DOI:** 10.35946/arcr.v38.1.08

**Published:** 2016

**Authors:** Tamara L. Wall, Susan E. Luczak, Susanne Hiller-Sturmhöfel

**Affiliations:** Tamara L. Wall, Ph.D., is a professor in the Department of Psychiatry at the University of California, San Diego, and associate chief of the Psychology Service at the Veterans Affairs San Diego Healthcare System, San Diego, California. Susan E. Luczak, Ph.D., is an associate research professor at the University of Southern California, Los Angeles, California. Susanne Hiller-Sturmhöfel, Ph.D., is senior science editor at *Alcohol Research: Current Reviews.*

**Keywords:** Alcohol dependence, alcohol use disorder (AUD), alcohol metabolism, alcohol-metabolizing enzymes, genetic factors, environmental factors, biological factors, gene variants, alcohol dehydrogenase (ADH), aldehyde dehydrogenase (ALDH), alleles, acetaldehyde, Asians, Caucasians, Africans, Asian-American, African-American

## Abstract

Gene variants encoding several of the alcohol-metabolizing enzymes, alcohol dehydrogenase (ADH) and aldehyde dehydrogenase (ALDH), are among the largest genetic associations with risk for alcohol dependence. Certain genetic variants (i.e., alleles)—particularly the ADH1B*2, ADH1B*3, ADH1C*1, and ALDH2*2 alleles—have been associated with lower rates of alcohol dependence. These alleles may lead to an accumulation of acetaldehyde during alcohol metabolism, which can result in heightened subjective and objective effects. The prevalence of these alleles differs among ethnic groups; ADH1B*2 is found frequently in northeast Asians and occasionally Caucasians, ADH1B*3 is found predominantly in people of African ancestry, ADH1C*1 varies substantially across populations, and ALDH2*2 is found almost exclusively in northeast Asians. Differences in the prevalence of these alleles may account at least in part for ethnic differences in alcohol consumption and alcohol use disorder (AUD). However, these alleles do not act in isolation to influence the risk of AUD. For example, the gene effects of ALDH2*2 and ADH1B*2 seem to interact. Moreover, other factors have been found to influence the extent to which these alleles affect a person’s alcohol involvement, including developmental stage, individual characteristics (e.g., ethnicity, antisocial behavior, and behavioral undercontrol), and environmental factors (e.g., culture, religion, family environment, and childhood adversity).

Epidemiological studies have demonstrated that drinking patterns and the prevalence of alcohol-related adverse consequences, including alcohol use disorder (AUD), differ substantially among racial/ethnic groups in the United States. For example, analyses comparing drinking patterns and their consequences among Whites, Blacks, Asians, and Hispanics found the following: Whites have the highest risk and Asians have the lowest risk of AUD among these ethnic groups; Hispanics have higher rates and Asians have lower rates of heavy drinking than do Whites; and Hispanics and Blacks are more likely to have health and social problems from drinking than are Whites and Asians (Chartier and Caetano 2010). Other studies have found subgroup differences within racial/ethnic groups for alcohol-related problems; for example, individuals of Korean ancestry have higher rates of AUD than those of Chinese ancestry (Helzer et al. 1990; Luczak et al. 2004).

These differences among racial/ethnic/ancestry groups result from a variety of biological, genetic, and environmental influences, some of which relate to the metabolism of alcohol and are explored in this article. Genes encoding several variants of alcohol-metabolizing enzymes are among the largest genetic associations with the risk for alcohol dependence (Li 2000). This article briefly reviews how alcohol is metabolized in the body and describes ethnic differences in some of the genes encoding the enzymes involved in alcohol metabolism, as well as the mechanism by which these genes are thought to give rise to differences in rates of alcohol dependence. The article also summarizes what is known about potential individual and environmental influences that may moderate the effects of these gene variants.

## Alcohol Metabolism

The key enzymes involved in alcohol metabolism in the liver are alcohol dehydrogenase (ADH) and aldehyde dehydrogenase (ALDH). ADH mediates (i.e., catalyzes) the oxidation of beverage alcohol (ethanol) into acetaldehyde. Acetaldehyde then is further metabolized by ALDH into acetate. These two reactions need to be properly coordinated in the body because accumulation of acetaldehyde can lead to heightened responses as well as unpleasant reactions, such as flushing, nausea, vomiting, hypotension, and/or rapid heartbeat (i.e., tachycardia). Variant forms of several ADH and ALDH enzymes exist and are encoded by an individual’s genes. These variants (i.e., alleles) produce enzymes with different properties, resulting in potential differences in the rates with which alcohol or acetaldehyde are metabolized. As a result, these variants also may influence a person’s response to alcohol, drinking behavior, and consequent risk of developing an AUD. People possessing certain ADH or ALDH alleles have significantly lower rates of alcohol dependence. The following sections review four of the best-studied ADH and ALDH variants—ADH1B*2 (rs1229984), ADH1B*3 (rs2066702), ADH1C*1 (rs698), and ALDH2*2 (rs671)—and their associations with a variety of alcohol-related factors or phenotypes. The table reports the allele frequencies of these genes in different populations.

### ADH Variants

To date, seven different ADH genes—*ADH1A, ADH1B, ADH1C, ADH4, ADH5, ADH6,* and *ADH7*—have been identified clustered together on the long arm of chromosome 4 (Edenberg 2007). Of these, the *ADH1A*, *ADH1B*, and *ADH1C* genes encode the majority of the ADH enzymes that metabolize alcohol in the liver. Several genome-wide association studies of alcohol dependence have found significant results in the region of chromosome 4q that includes the ADH gene cluster in a variety of ethnically diverse samples (e.g., Gelernter et al. 2014). The ADH gene with the largest effect size with alcohol dependence is *ADH1B*. Significant associations have been found for the ADH1B*2 allele and alcohol dependence in Asian populations (Li et al. 2012*a*; Luczak et al. 2006*a*), as well as in European and African-American populations (Bierut et al. 2012; Whitfield 1997, 2002). Whitfield (2002) found that Europeans with one ADH1B*2 allele were about half as likely (odds ratio [OR] = 0.47) to be alcohol dependent as individuals without this genetic variant (ADH1B*1/*1 genotype). In a large meta-analysis of Asian, European, African, Hispanic, and Native-American samples, individuals with an ADH1B*2 allele overall were about half as likely to be alcohol dependent as those without this genetic variant (OR = 0.49) (Li et al. 2012*a*). The protective association is also greater for individuals with two ADH1B*2 alleles (Li et al. 2012*a*; Luczak et al. 2006*a*). When subgroup analyses were conducted, the associations were larger in Asian populations (Li et al. 2012*a*). This is likely a result of the combined effects of the ADH1B*2 and ALDH2*2 alleles, as expanded upon below (Luczak et al. 2006*a*).

A second *ADH1B* gene variant, the ADH1B*3 allele, has been related to lower rates of alcohol dependence in many but not all association studies (Edenberg 2007; Edenberg et al. 2006, 2010; Ehlers et al. 2001, 2007; Gizer et al. 2011; Luo et al. 2006; Wall et al. 1997*a*). Significant associations for the ADH1B*3 allele and alcohol dependence primarily have been found in individuals of African ancestry where this genetic variant is most prevalent (Edenberg et al. 2006; Luo et al. 2006).

A variant of the *ADH1C* gene, the ADH1C*1 allele, also has been well studied with respect to alcohol dependence, but the results have been inconsistent because of limited sample sizes, ethnic variation, and the close proximity of the *ADH1B* and *ADH1C* genes. Some studies showed that ADH1C*1 and ADH1B*2 are in linkage disequilibrium, suggesting that associations of ADH1C*1 with alcohol dependence may be attributed to correlation with ADH1B*2 (Borras et al. 2000; Chen et al. 1999*a*; Osier et al. 1999). A large meta-analysis of Asian, European, African, and Native-American samples found that individuals with an ADH1C*1 allele overall were about one-third as likely to be alcohol dependent as those without this genetic variant (OR = 0.66) and also demonstrated a larger effect (OR = 0.48) in Asian populations (Li et al. 2012*b*). Furthermore, linkage disequilibrium analyses located the *ADH1C* gene in a different haplotype block than the *ADH1B* gene, suggesting the associations may be independent of one another, even though the two genes are close together.

The proposed mechanism by which these ADH alleles lead to lower rates of alcohol dependence relate to differences in the characteristics of the enzymes that they ultimately encode. The ADH1B*2 and ADH1B*3 alleles are thought to encode enzymes that oxidize ethanol at an increased rate compared with enzymes encoded by the more common ADH1B*1 allele, resulting in faster acetaldehyde production. Because this increased production may lead to the accumulation of acetaldehyde and potentially more intense and/or unpleasant alcohol reactions (e.g., a flushing response), people carrying these alleles may be less likely to drink alcohol, particularly at high levels, and accordingly they also may be less likely to develop an AUD (Wall 2005; Wall et al. 2013). Similarly, the ADH1C*1 allele is thought to encode an enzyme that accelerates the conversion rate of alcohol into acetaldehyde relative to the ADH1C*2 allele and thus may lead to acetaldehyde buildup after alcohol consumption, thereby promoting reduced alcohol consumption and ultimately protection against AUD (Li et al. 2012*b*).

The findings assessing this proposed mechanism of action—that *ADH1B* and *ADH1C* variations reduce alcohol dependence risk through elevated acetaldehyde levels, heightened responses to alcohol, and reduced drinking—have been inconsistent. ADH1B*2, ADH1B*3, and ADH1C*1 have not been associated with elevations in acetaldehyde, although acetaldehyde is difficult to measure in the low concentrations expected from these alleles. Many but not all studies have found that ADH1B*2 is associated with increased sensitivity to alcohol (i.e., increased flushing and associated symptoms; see Wall et al. 2013 for review). The ADH1B*3 allele has been associated with a faster rate of alcohol elimination and a more intense response to alcohol in individuals of African ancestry (McCarthy et al. 2010; Thomasson et al. 1995).

### ALDH Variants

The acetaldehyde generated by the ADH-mediated oxidation of ethanol is further oxidized by two main ALDH enzymes—ALDH1 and ALDH2—encoded by different genes. With regard to ALDH, the ALDH2*2 allele has shown the largest association with alcohol dependence. A meta- analysis of studies of Asian samples (Luczak et al. 2006*a*) indicated that having one ALDH2*2 allele was associated with a four- to fivefold reduction in alcohol dependence (OR = 0.22), and having two ALDH2*2 alleles was associated with an eight- to ninefold reduction in alcohol dependence (OR = 0.12). This meta-analysis also examined the effect of ALDH2*2 and ADH1B*2 alleles in combination on the risk for alcohol dependence (Luczak et al. 2006*a*). In ALDH2*1/*1 individuals (i.e., ALDH2*1 homozygotes), one ADH1B*2 allele was associated with about one-fourth (OR = 0.26) and two ADH1B*2 alleles were associated with about one-fifth (OR = 0.20) the risk of alcohol dependence compared with individuals with no ADH1B*2 alleles. In ALDH2*1/*2 individuals (people who carry one ALDH2*2 allele and one ALDH2*1 allele; i.e., who are heterozygous), one ADH1B*2 allele was associated with about one-sixth (OR = 0.17) and two ADH1B*2 alleles were associated with about one-eleventh (OR = 0.09) the risk of alcohol dependence compared with individuals with no ADH1B*2 alleles. These results suggest both *ALDH2* and *ADH1B* each contribute unique protective effects on alcohol dependence, and the level of protection may be even stronger in conjunction than alone (i.e., a gene × gene interaction exists).

A similar mechanism of action has been proposed for how ALDH2*2 results in lower rates of alcohol dependence (Wall 2005; Wall et al. 2013). According to this model, ALDH2*2 encodes a deficient protein subunit that has low or no activity. As a result, acetaldehyde generated by the actions of ADH cannot be readily metabolized and accumulates in the body. Consistent with this assumption, in vitro and in vivo studies have demonstrated that compared with the enzyme activity generated in cells or organisms homozygous for ALDH2*1 (i.e., ALDH2*1/*1 genotype), those who are heterozygous show only 12 to 20 percent of the enzyme activity and elevated acetaldehyde levels, and those who are homozygous for ALDH2*2 show no enzyme activity and even higher acetaldehyde levels (Bosron and Li 1986; Wall et al. 1997*b*). Consequently, people who are homozygous for ALDH2*2 experience acetaldehyde buildup even after consuming only small amounts of alcohol. As a result, these individuals rarely consume large amounts of alcohol, and there are very few documented cases of people with this genotype having alcohol dependence (Chen et al. 1999*b*; Luczak et al. 2004).

Because of the accumulation of acetaldehyde, people carrying the ALDH2*2 allele are thought to experience heightened responses to alcohol. This has been confirmed in self-report and alcohol-challenge studies. Thus, in self-report studies ALDH2*2 has been related to indicators of alcohol sensitivity, such as alcohol-induced flushing and other symptoms (e.g., nausea, headaches, and palpitations). Similarly, numerous alcohol-challenge studies found that people who are heterozygous for ALDH2*2 experience flushing as well as changes in pulse rate, hormone levels, psychomotor performance, and neurophysiological reactivity compared with people homozygous for ALDH2*1 who had the same blood alcohol concentrations. People who are homozygous for ALDH2*2 experience even more intense subjective and objective reactions to alcohol (see Wall et al. 2013).

As a result of this heightened sensitivity to alcohol, people with the ALDH2*2 allele may have lower positive and higher negative expectancies about alcohol’s effects. Alcohol expectancies are thought to be mediators between the biological factors that determine the physiological consequences of alcohol consumption and a person’s actual alcohol use. Thus, people who are highly sensitive to alcohol’s unpleasant effects because they carry the ALDH2*2 allele may be less likely to drink because they do not expect alcohol to have pleasant, reinforcing effects and instead may expect it to have unpleasant, aversive ones. Several studies examining the association between ALDH2*2 and alcohol expectancies support this hypothesis. Two studies (McCarthy et al. 2000, 2001) found that ALDH2*2 was associated with reduced positive expectancies but was unrelated to negative expectancies. In another analysis (Hendershot et al. 2009*b*), people with ALDH2*2 alleles reported greater negative expectancies and thought that alcohol had greater physiological effects than did people without the allele.

The greater sensitivity to alcohol and the resulting altered alcohol expectancies then are likely to lead to lower rates of drinking and of heavy drinking. Thus, several studies have found that people with one ALDH2*2 allele showed lower quantity and frequency of alcohol use and engaged in less binge drinking than did people without this allele; the presence of two ALDH2*2 alleles exacerbated these effects (see Wall et al. 2013). Reduced consumption, in turn, leads to fewer alcohol-related adverse consequences, as indicated by lower scores on questionnaires measuring hazardous alcohol use and alcohol-related problems (Hendershot et al. 2009*a*, 2011). Similarly, hangovers and blackouts as consequences of heavy drinking also are inversely associated with ALDH2*2 (Luczak et al. 2006*b*; Wall et al. 2000). A longitudinal study found that ALDH2*2 changes the association between alcohol consumption and problems over time, with ALDH2*2 group differences in alcohol-related problems fully accounted for by differences in frequency of binge drinking (Luczak et al. 2014).

Similar to the results from meta- analyses showing that the *ALDH2* and *ADH1B* genes may have an interactive effect on alcohol dependence (Luczak et al. 2006*a*), some self-report and alcohol-challenge data in Asians suggest that the effects of ADH1B*2 may be stronger in individuals with ALDH2*1/*2 genotype (e.g., Chen et al. 1999*b*; Cook et al. 2005; Luczak et al. 2006*b*; Takeshita et al. 1996, 2001). For example, in one study of Asians who carried the ADH1B*2 allele, a heightened sensitivity to alcohol was reported only if they also carried the ALDH2*2 allele, whereas no increase in sensitivity was reported by people carrying ADH1B*2 in combination with only ALDH2*1 alleles (Luczak et al. 2011). Similarly, an alcohol-challenge study only found an increased response to alcohol in people with ADH1B*2 who also were heterozygous for ALDH2*2 (Cook et al. 2005). These results suggest that the effects of ADH1B*2 may be felt more strongly in Asians who already have some heightened sensitivity to alcohol from possessing one ALDH2*2 allele, but additional research is needed to confirm these findings.

## Ethnic Differences in Prevalence of *ADH1B*, *ADH1C*, and *ALDH2* Alleles

### Prevalence of ADH1B and ADH1C Alleles

The ADH1B*2 allele is found in 80 percent or more of northeast Asians (i.e., Chinese, Japanese, and Koreans) and about 50 percent of Russians and Jews, but only in 10 percent or less of Caucasians of European ancestry (Goedde et al. 1992; Osier et al. 2002). However, within the large Asian ethnic group, variations in the prevalence of the ADH1B*2 allele exist among subpopulations (Eng et al. 2007).

The ADH1B*3 allele is found predominantly in people of African ancestry (about 30 percent) and in much lower prevalence in certain Native Americans (i.e., Mission Indians), likely because of admixture (Bosron et al. 1983; Edenberg et al. 2006; Wall et al. 1997*a*, 2003). This allele rarely has been found in Asians and Whites.

The ADH1C*1 allele varies substantially across different populations. It is highly prevalent in Asian and African groups (80 percent or more) and lower in Caucasians of European ancestry (about 50 percent) (Eng et al. 2007; Li et al. 2012*b*).

### Prevalence of ALDH2 Alleles

ALDH2*2 is found almost exclusively in northeastern Asian populations, albeit with varying prevalences among different Asian ethnicities (see Eng et al. 2007). For example, among Han Chinese, overall approximately one-third of individuals possess at least one ALDH2*2 allele, with different studies determining prevalence ranging from 20 to 47 percent of participants. In contrast, ALDH2*2 was much less commonly found among Chinese and Taiwanese natives. Studies of Japanese identified prevalence rates of 41 to 52 percent for the ALDH2*2 allele, whereas analyses of Koreans found ALDH2*2 prevalence of 29 to 37 percent. In other Asian ethnicities (e.g., Thais), the ALDH2*2 allele is much less common and is found only in 10 percent or less of individuals. In all cases, only a small proportion of the individuals were homozygous for this allele (about 5 percent); most were heterozygous (Eng et al. 2007).

## Moderators of the Effects of ADH1B*2 and ALDH2*2

Although the studies described above demonstrate that *ADH1B* and *ALDH2* variants influence the risk of AUD, it also is clear these genes and their alleles do not act in isolation. The effects of the ADH1B*2 allele on a person’s risk of AUD also depend on the person’s *ALDH2* genotype. Thus, Asians who carry the ALDH2*2 allele show a greater protective effect (i.e., a lower risk of alcohol dependence) from the ADH1B*2 allele than do people who only carry the functional ALDH2*1 allele (Luczak et al. 2006*a*). However, numerous additional factors may influence the extent to which ALDH2*2 and ADH1B*2 affect a person’s risk of alcohol involvement and AUD. Even the design of the studies assessing the associations between genotypes and AUD risk may influence the results. Thus, results from a meta-analysis study found that both the diagnostic system used in a study and the recruitment strategy used to identify study participants moderated the effects of ALDH2*2 on risk of alcohol dependence (Luczak et al. 2006*a*). For example, studies that used the more stringent criteria of the *International Code of Diseases, 10^th^* *Edition* (ICD–10) to establish an AUD diagnosis rather than the less stringent criteria of the *Diagnostic and Statistical Manual of Mental Disorders, 3rd Edition, Revised* (DSM–III–R) revealed a greater protective effect of ALDH2*2. Similarly, studies in which participants were recruited from treatment settings showed greater protective effects of ALDH2*2 than did studies involving recruitment of community samples, Thus, these findings demonstrate the importance of methodological issues that must be considered when examining the influence of moderators of gene effects. Only by accounting for these potential moderators will researchers be able to further understand the influences of these alleles and their interactions with other variables on alcohol-related behaviors and the risk of AUD.

Other possible moderators of these gene effects include the following:

- Developmental stage;
- Individual characteristics, such as ethnicity, antisocial behavior, and behavioral undercontrol; and
- Environmental factors, such as culture, religion, family environment, and childhood adversity.

These factors are discussed in the following sections. Because ALDH2*2 has the largest effect on alcohol dependence and because it is found almost exclusively in Asian populations, most of this discussion will focus on this gene and these ethnic groups.

### Developmental Stage

The magnitude of ALDH2*2 effects on alcohol use phenotypes has been shown to change over the course of development. In particular, associations of ALDH2*2 with alcohol- related measures become stronger over the course of adolescence and young adulthood as alcohol use increases (Doran et al. 2007; Irons et al. 2007, 2012; Luczak et al. 2014). These findings are consistent with twin studies and studies of other candidate genes where genetic influences on alcohol phenotypes increase with age (Dick et al. 2006; Rose and Dick 2005).

Furthermore, although ALDH2*2 protects against the development of alcohol dependence, the protection is not complete. In the presence of alcohol dependence or at lower levels of alcohol use, individuals with ALDH2*2 alleles are more vulnerable to alcohol-related pathologies—particularly head and neck cancers, but also liver disease, pancreatitis, and Alzheimer’s disease—consistent with a role of acetaldehyde in the pathogenesis of organ damage (Brennan et al. 2004; Brooks et al. 2009; Hao et al. 2011; Lewis and Smith 2005; Yang et al. 2010; Zhang et al. 2010; Zintzaras et al. 2006). Thus, the influence of ALDH2*2 seems to change over the course of drinking; that is, ALDH2*2 is protective at one stage of alcohol use (i.e., progression to heavy drinking) but becomes a risk factor at another stage (i.e., progression to alcohol-related medical problems). Prospective studies are needed to determine how gene effects may change over the lifespan.

## Individual Characteristics

### Ethnicity

A study comparing Korean Americans and Chinese Americans examined whether differences in the prevalence of the ALDH2*2 allele mediated ethnic differences in AUD and whether the effect of ALDH2*2 was moderated by ethnicity (Luczak et al. 2004). These analyses found that ALDH2*2 was a significant mediator of protection against alcohol dependence across different ethnic groups. However, no significant interaction existed between ALDH2*2 and ethnicity. Another study, in contrast, found an interaction between ALDH2*2, ethnicity (i.e. Korean vs. Chinese), and alcohol dependence (Luczak et al. 2001). Chinese with an ALDH2*2 allele were about one-quarter as likely to be alcohol dependent as those without the allele, whereas among the Koreans those with ALDH2*2 were half as likely to be alcohol dependent. This finding suggests that ALDH2*2 may have a stronger protective effect in Chinese than in Koreans. However, additional studies are needed to further explore this issue to conclusively determine the interplay between ALDH2*2 and ethnicity, as well as other factors that might underlie ethnic differences.

### Antisocial Behavior

Antisocial behavior and conduct disorder (CD) consistently have been identified as risk factors for alcohol use and AUD (see Krueger et al. 2002; Waldman and Slutske 2000). In both genders, symptoms of antisocial behavior and CD precede alcohol-related problems (Disney et al. 1999; Slutske et al. 1998). The prevalence of antisocial behavior as indicated by a diagnosis of antisocial personality disorder (ASPD) and CD differs among men and women and also shows racial/ethnic differences. In all populations studied, the prevalence for these conditions is significantly higher among men than among women (e.g., Lee et al. 1990; Luczak et al. 2004). Ethnic differences have been demonstrated particularly among Asian populations. For example, the rates of ASPD were substantially higher among South Koreans (1.6 percent) (Lee et al. 1990) than among Taiwanese (0.1 to 0.2 percent) (Hwu et al. 1989). Similarly, the prevalence of CD was higher among Korean-American college students (29 percent of men and 2 percent of women) than among Chinese-American college students (9 percent of men and 2 percent of women) (Luczak et al. 2004).

Several studies have analyzed whether differences in prevalence of protective alleles of alcohol-metabolizing enzymes and ASPD/CD could account for differences in the prevalence of AUD in different populations. A study assessing the relationship between ALDH2*2, CD, and alcohol dependence in Korean Americans and Chinese Americans found that although CD was a significant mediator of alcohol dependence, no significant interaction existed between CD and ALDH2*2. In other words, both ALDH2*2 and CD influenced the risk of alcohol dependence, but these effects were independent of each other (Luczak et al. 2004). Other studies, however, have suggested that ASPD might interact with ALDH2*2 to influence alcohol dependence. A study comparing *ALDH2* and *ADH1B* allele status in Taiwanese with and without ASPD and/or alcohol dependence found that ALDH2*2 showed reduced association with alcohol dependence in people with ASPD compared with people without ASPD. ADH1B*2 also no longer showed any association with alcohol dependence in antisocial alcoholics (Lu et al. 2005). Another study found that the prevalence of ASPD was higher in alcoholics with the ALDH2*2 allele than in alcoholics without this allele (Iwahashi 1995). These findings suggest that the protective effects of ALDH2*2 may be less strong in people with more antisocial behavior.

### Behavioral Undercontrol

One of the personality traits known to predict alcohol and other drug use and abuse is behavioral undercontrol, a personality trait characterized by impulsivity, sensation seeking, and disinhibition (Sher et al. 2000). It also can explain, at least in part, the association between CD and AUD discussed above—that is, people with behavioral undercontrol also are more likely to be diagnosed with CD (Slutske et al. 2002). Researchers have investigated whether the increase in AUD risk conferred by behavioral undercontrol interacts with the reduction in risk conferred by ALDH2*2. One study (Doran et al. 2007) examined whether *ALDH2* status and the levels of behavioral undercontrol influenced the risk of binge drinking over a 2-week period in 18- to 29-year-old college students. The study found that, as expected, ALDH2*2 reduced the risk of binge drinking, whereas behavioral undercontrol increased binge-drinking frequency. However, behavioral undercontrol did not seem to moderate the effects of ALDH2*2; instead, the effects of both factors were additive. This finding may be explained by the fact that behavioral undercontrol seems to act primarily at the level of alcohol use initiation (i.e., people with high levels of impulsivity and sensation seeking may be particularly likely to try alcohol and other drugs). In contrast, ALDH2*2 influences not alcohol use initiation but continued use (i.e., people with ALDH2*2 are less likely to continue using alcohol because they experience more intense effects).

## Environmental Factors

### Culture

Cultural influences, such as societal beliefs regarding alcohol use, which are shaped by traditions, religious beliefs, and other philosophies widely acknowledged within a society, also shape drinking behaviors. For example, both Chinese and Korean cultures are influenced by Confucian philosophy, which emphasizes drinking in moderation (Bond and Hwang 1986; Cheng 1980). In addition, however, in Korean culture it also is important, especially for men, to socialize and drink heavily, which may result in greater acceptance of heavy drinking and alcohol problems (Cho and Faulkner 1993; Higuchi et al. 1996; Park et al. 1998*a,b*,*b*). Such cultural differences may contribute to the observed higher prevalence of AUD in people of South Korean heritage compared with those of Chinese or Taiwanese heritage (Helzer et al. 1990; Luczak et al. 2004). However, as mentioned previously, differences in the prevalence of ALDH2*2 and ADH1B*2 between different Asian ethnic groups also may account for at least part of the difference in AUD prevalence.

Further support for the relationship between culture and drinking behavior comes from observations that changes in cultural influences over time also may be followed by changes in drinking behaviors. Such developments, which have been observed in several Asian countries, also may moderate the influence of biological protective factors such as ALDH2*2. For example, a Japanese study found that between 1979 and 1992, when alcohol consumption became more culturally accepted and social pressure to drink increased, the proportion of Japanese patients who received treatment for alcohol dependence and carried the ALDH2*2 allele increased from 2.5 percent to 13 percent, indicating that the protective effects of ALDH2*2 had declined (Higuchi et al. 1994). Along the same lines, increasing acculturation of Asian Americans to American culture led to more heavy drinking and binge drinking (Hendershot et al. 2005). However, the extent of this effect was influenced by ethnicity. Thus, greater levels of acculturation in the United States may increase binge-drinking risk among people of Chinese origin but not among those of Korean origin.

### Religion

Higher levels of religious behavior (e.g., commitment, affiliation, and service attendance, primarily with Christian religions) have been associated with lower alcohol use and related problems in the United States (e.g., Cochran et al. 1988; Midanik and Clark 1994; Wechsler et al. 1998). Similar analyses have been conducted with Asian and Asian-American populations, with different results depending on the population studied. Thus, whereas religious affiliation and involvement, particularly with Protestant denominations, was related to lower rates of alcohol involvement among Korean Americans (Lubben et al. 1989), the findings were inconsistent for Chinese Americans (Chi et al. 1988, 1989). In another study, religious affiliation as measured by service attendance was related to lower rates of binge drinking in Koreans regardless of their religion; among Chinese, however, such a relationship was found only among those affiliated with Western religions (Luczak et al. 2003).

Because twin studies have identified gene–environment interactions of religiosity with alcohol use behavior (Heath et al. 1999; Koopmans et al. 1999), researchers also have investigated potential interactions with ALDH2*2 status. These analyses found that religiosity moderated the association of ALDH2*2 with binge drinking (Luczak et al. 2003). Specifically, religious service attendance was related to binge drinking only in people homozygous for ALDH2*1, but not in those with at least one ALDH2*2 allele, suggesting that the protective effect of ALDH2*2 may be less strong in people with higher levels of religiosity.

### Family Environment

Adoption studies can be especially informative for disentangling genetic influences from those of social environment. In particular, studies of adoptees can help determine if effects may be due to genetic factors or modeling behavior in the adoptive family environment. A study of adopted adolescents and young adults of Asian descent found that the effect of ALDH2*2 was moderated by environmental influences of parental alcohol use and misuse as well as sibling alcohol use. Specifically, high parental alcohol use and misuse reduced the protective effect of ALDH2*2 on alcohol phenotypes, whereas low parental alcohol use and misuse enhanced the effect of the allele (Irons et al. 2012). In a similar fashion, sibling alcohol use also appeared to moderate the effect of ALDH2*2 on an adoptee’s drinking behavior (Irons et al. 2007).

### Childhood Adversity

Many but not all studies have shown that exposure to adverse events in childhood, such as sexual, emotional, and physical abuse, is a risk factor for developing an AUD in adulthood (Keyes et al. 2011). In a sample of Israeli adults with a relatively high prevalence of the ADH1B*2 allele (47 percent either heterozygous or homozygous), a history of childhood adversity moderated the influence of ADH1B*2 on alcohol-related phenotypes (Meyers et al. 2015). There was a stronger effect of ADH1B*2 on AUD severity and the maximum number of drinks consumed in a day in individuals who had a history of childhood adversity compared with those who did not. Thus, ADH1B*2 seems to exert a stronger effect in individuals whose risk for drinking is increased by their childhood adversity, although longitudinal studies are needed to confirm this finding.

## Conclusions

Variations in the alcohol-metabolizing enzymes ADH and ALDH and the genes encoding them are associated with alcohol-related behaviors and the risk of AUD. In particular, the ADH1B*2, ADH1B*3, ADH1C*1, and ALDH2*2 alleles have shown protective associations with alcohol dependence. The ADH1B*2, ADH1C*1, and ALDH2*2 alleles have high prevalence in Asian populations and the ADH1B*3 and ADH1C*1 alleles in African populations, which may contribute to the differences in AUD prevalence observed among larger racial groups (i.e., Whites, Blacks, and Asians). Moreover, the prevalence of these alleles varies among different Asian subpopulations and may account at least in part for the different rates of AUD among those populations.

However, it also is clear that these alleles alone cannot explain all the differences in AUD prevalence between racial and ethnic groups; individual and environmental factors also play a role. In studies of Asian populations, some of these factors demonstrate additive effects to those imparted by ADH1B*2 and ALDH2*2. In other cases, however, these additional factors interact with and moderate the effects of these alleles. In addition, a gene–gene moderating effect appears to exist between ADH1B*2 and ALDH2*2, such that among people of Asian descent the effects of ADH1B*2 may be larger in those who also carry ALDH2*2. Further exploration of the interactions between various genetic, individual, and environmental factors influencing drinking behavior and thus risk of AUD is necessary to fully understand how drinking behavior is shaped across developmental stages, which individual characteristics place people at risk for alcohol-related problems or AUD, when and where individuals are at most or least risk, and how preventive measures and interventions can reduce risk.

## Figures and Tables

**Table:** Gene Frequencies of Specific Alleles of the Genes Encoding Alcohol Dehydrogenase (ADH) and Aldehyde Dehydrogenase (ALDH) in Different Ethnic Populations

Allele	rs Number	Frequency in Different Populations		
ADH1B*2	***rs1229984***		**A allele**	**G allele**
		European	0.000–0.008	0.992–1.000
		Asian	0.739–0.771	0.229–0.261
		Sub-Saharan African	0.000	1.000
		African American	0.000	1.000

ADH1B*3	***rs2066702***		**C allele**	**T allele**
		European	1.000	0.000
		Asian	1.000	0.000
		Sub-Saharan African	0.500–0.783	0.217–0.500
		African American	0.733	0.267

ADH1C*1	***rs698***		**C allele**	**T allele**
		European	0.523–0.527	0.473–0.477
		Asian	0.927–0.975	0.025–0.073
		Sub-Saharan African	0.938–0.958	0.042–0.062
		African American	0.800	0.200

ALDH2*2	***rs671***		**C allele**	**T allele**
		European	0.000	1.000
		Asian	0.110–0.282	0.718–0.890
		Sub-Saharan African	0.000	1.000
		African American	0.000	1.000

SOURCE: dbSNP Database (www.ncbi.nlm.nih.gov/snp).
